# Declining Abundance and Variable Condition of Fur Seal (*Arctocephalus forsteri*) Pups on the West Coast of New Zealand’s South Island

**DOI:** 10.3390/ani16010121

**Published:** 2025-12-31

**Authors:** Alasdair A. Hall, Don Neale, Jim Roberts, B. Louise Chilvers, Jody Suzanne Weir

**Affiliations:** 1 Hall Marine Consulting, Wellington 6021, New Zealand; 2 Department of Conservation, Hokitika 7810, New Zealand; 3 Anemone Consulting Limited, Wellington 6022, New Zealand; 4 Wildbase, School of Veterinary Science, Massey University, Private Bag 11 222, Palmerston North 4442, New Zealand; 5 Department of Conservation, Kaikōura 7300, New Zealand

**Keywords:** *Arctocephalus forsteri*, body condition, population health, fisheries bycatch, fur seal, population decline

## Abstract

New Zealand fur seals (*Arctocephalus forsteri*) were severely exploited by historical hunting. However, recently assessed colonies in New Zealand are mostly thought to be growing or stable. The exceptions are three colonies (Wekakura Point, Cape Foulwind and Taumaka Island) on the West Coast of the South Island (‘WCSI’), previously documented as in decline. We used mark-recapture and morphometric data to update understandings of pup abundance and condition at these colonies. Pup abundance has continued to decline. In 2025, 186 (95% CI = 178–194) pups were estimated at Wekakura Point, 131 (95% CI = 122–140) at Cape Foulwind and 566 (95% CI = 555–577) at Taumaka Island, representing declines of 83%, 71% and 61% from the respective maxima in the 1990s. Rates of decline have slowed at Wekakura Point and Cape Foulwind since 2016 but have increased at Taumaka Island. Pup condition demonstrated substantial interannual variation. Cape Foulwind pups had the greatest average mass and body condition index score, followed by Wekakura Point and then Taumaka Island. There have been consistencies between years of particularly low pup abundance and condition across the colonies, suggesting common stressors; however, there are likely also some localised factors. Emerging diseases and marine environmental change are evaluated as potential drivers.

## Simple Summary

New Zealand fur seals are generally thought to be increasing in numbers around New Zealand, although relatively few colonies are monitored regularly. We provide updates on pup abundance and condition from three colonies (Wekakura Point, Cape Foulwind and Taumaka Island) on the West Coast of New Zealand’s South Island where pup abundance has been declining. We found that pup numbers have continued to fall, and that pup condition has varied significantly through time. From the maximum numbers of pups born at each colony in the 1990s, Wekakura Point has declined by 83%, Cape Foulwind by 71% and Taumaka Island by 61% to 2025. There have been consistencies between the colonies in the pup abundance trends, as well as correlations between years of low pup abundance and poor pup condition. Pup mortality has been substantially higher at Taumaka Island than at either Wekakura Point or Cape Foulwind. Given the trends observed, and previous research into the impacts of climate change on marine ecosystems in this region, it seems likely that environmental change is influencing fur seal pup production and condition. However, this mechanism and other potential influences on the observed population trends require further study.

## 1. Introduction

As with other pinniped species globally, New Zealand fur seal/kekeno (*Arctocephalus forsteri*) were subjected to uncontrolled historical harvesting which drove them to the brink of extinction [1,2,3,4,5,6]. Although the cessation or reduction in commercial sealing in most countries has allowed for recoveries [3,7,8,9,10,11], in recent decades previously increasing pinniped populations have experienced declining abundance; for example, Australian fur seal (*Arctocephalus pusillus doriferus*) and Antarctic fur seal (*Arctocephalus gazella*) [11,12,13]. Among the confirmed and potential causes for declines in pinniped populations are emerging diseases [14,15,16], long-term environmental change as well as stochasticity [17,18,19,20] and competition from and bycatch in commercial fisheries [21,22,23].

Determining causation for pinniped population change is complex [13,24,25]. Longitudinal monitoring offers the ability to detect real population change over time [24,26,27] with greater reliability than short-term studies, which may struggle to account for interannual variation in parameters such as survival and fecundity [28,29]. The insights gathered from regular sampling also provide opportunities to move beyond detecting change into interpreting causation, which can inform management action [30,31,32,33]. In contrast, when population data are missing, the implementation of appropriate mitigation responses can be impeded [23,30,34]. Longitudinal monitoring programmes that focus on sentinel species have benefits beyond conserving or managing the programme’s subject. For example, detection of high mercury levels in bottlenose dolphins (*Tursiops truncatus*) in Florida have raised concerns about human consumption of seafood derived from the same locale [35]. Similarly, monitoring relatively abundant species can provide important information for understanding potential risks to rarer taxa that occupy comparable ecological or geographic niches [36,37]. While longitudinal monitoring programmes are typically resource intensive [38,39] the advent and continual improvement of remote sensing technologies, such as unmanned aerial vehicles (UAVs) and satellite imagery, can reduce these costs [40].

**Figure 1:**
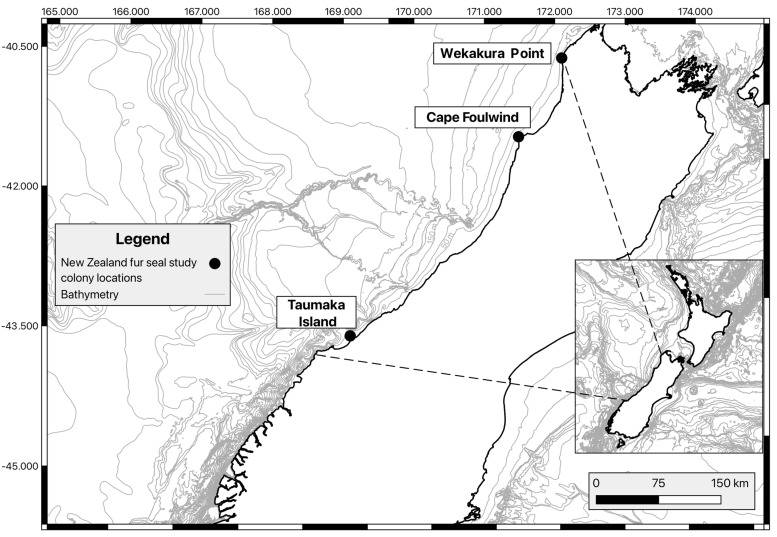
Locations of the New Zealand fur seal colonies (Wekakura Point, Cape Foulwind and Taumaka Island) on the West Coast of New Zealand’s South Island where pups have been surveyed in most years between 1991/92 and 2025.

Fur seal population monitoring has been ad hoc or non-existent for large areas of New Zealand [41]. A study of three colonies (Wekakura Point, Cape Foulwind and Taumaka Island) on the West Coast of New Zealand’s South Island (‘WCSI’) (Figure 1) represents the only multi-decadal monitoring programme for fur seals in New Zealand where both pup production and condition assessments are conducted. In otariids, pup production is used as a proxy for population abundance, as pups are confined to land until weaning and are instantly recognisable [42,43,44]. As female otariids are central-place income breeders, pup production is sensitive to changes in the local marine environment that impact prey abundance and distribution, and other factors that can impact female mortality [45,46,47]. Therefore, consistently executed pup production monitoring can enable detection of change in local marine ecosystems. Similarly, pathogens may impact pinniped reproductive cycles [20,48], meaning consistent monitoring may also be able to detect evidence for the presence of emerging diseases. Pinniped pup condition depends on a range of factors including female foraging efficiency [19,49]—itself dependent on both female experience [47,50] and the reliability of prey sources [18,51]—as well as female condition [52,53] and pathogen presence within the population [54,55,56]. Thus, monitoring pup condition can provide insights into both population health and female foraging efficiency. Further, as pup condition impacts survival both pre- and post-weaning [29,56], population-level changes in pup body condition may impact future population trajectories.

The WCSI New Zealand fur seal monitoring programme was established following concerns about high fur seal bycatch rates in the WCSI commercial hoki (*Macruronus novaezelandiae*) middle-depth trawl fishery in 1989 and 1990, and a previous report [57] described substantial declines in pup numbers over the period 1990/1991–2015/2016 (henceforth, breeding seasons are referred to by later year, e.g., 1991–2016) for all three study colonies. From their respective recorded maxima in the early/mid-1990s, by 2016 Wekakura Point had declined 79%, Cape Foulwind 69% and Taumaka Island by 34%. All colonies experienced abrupt drops in pup abundance in 1999–2000, and years with low pup masses corresponded with years where pup production estimates sharply declined [57].

Since 2016, another four seasons (2018, 2020, 2023 and 2025) of pup abundance and condition data have been collected at the WCSI colonies. Here, we use these data to update estimates of annual pup abundance trends at the only fur seal colonies in New Zealand known to be in long-term decline. We also use all the available pup morphometric data (1991–2025) to assess trends in pup condition using metrics comparable with previous fur seal studies [32,43].

Our objectives were to:

1. Establish whether previously observed trends in New Zealand fur seal pup abundance and condition have continued.
2. Examine the comparability of trends at the three study breeding colonies on the WCSI.
3. Provide recommendations for future research to determine the causes of the observed population trends.

## 2. Methods

### 2.1. Study Sites

Wekakura Point and Cape Foulwind are both on New Zealand’s mainland, while Taumaka Island is an island of approximately 20 ha, located ~5 kilometres offshore, and is part of the Open Bay Islands group (Figure 1). Cape Foulwind is accessible on foot by authorised personnel, while Wekakura Point and Taumaka Island are accessed by helicopter.

### 2.2. Live Pup Abundance Estimates 2018–2025

Live pup abundance estimates between 2018 and 2025 were derived from mark-recapture methods [42,43,44,58]. Fieldwork was undertaken as close as possible to the final week of January in each assessment year—a period when all pups in each breeding season had been born, after large territorial bulls have left the colonies and before pups are proficient swimmers and may no longer be confined to land [43,44,59]. There were, however, some deviations from this timeframe (Table 1).

Up to and including 2020, pups were marked with Allflex button tags inserted 15 mm from the trailing edge of each foreflipper. From 2023, pups were marked by trimming a small patch of outer fur on their heads [43,59,60]. Between 1991 and 2016, mark-recapture took place in most summer breeding seasons. Since 2016, assessments have occurred in 2018, 2020, 2023 and 2025. Over the study, the numbers of pups marked have decreased substantially due to declining colony sizes, as the aim of marking was to mark > 50% of the potential numbers of pups produced in a given season. Effort was made to spread marking consistently throughout each colony. The areas covered at Wekakura Point and Cape Foulwind have varied somewhat through time (K. Simister, pers. comm.), and to allow for this during analyses, data from the “Block Field” sector of Cape Foulwind were excluded from abundance analyses in this report.

Typically, recaptures commenced the day after marking was completed, allowing sufficient time for unmarked and marked pups to mingle [43,44,59,60]. Given the short period of time between marking and recapture, tag loss was assumed to be zero.

Occasionally, recaptures were delayed due to weather or personnel constraints (Table 1). During recapture counts, if a marked vs. unmarked designation could not be made confidently, an individual was discounted. Recapture methodologies differed between colonies due to variation in accessibility and terrain but were standardised by colony. At Wekakura Point, recaptures were made by a line of people traversing the colony and adjoining coastal zone on foot; at Cape Foulwind, recapture counts were made by a single person from selected clifftop vantage points; and at Taumaka Island recapture counts were made by a single person walking the length of the colony area in which marking was performed. For each marking session, there were typically five recapture counts, except at Taumaka Island in 2018 when only three were conducted. Personnel conducting the recapture counts changed between 2018 and 2023 at Cape Foulwind (K. Simister, pers. comm.), and in 2016 and 2018 at Taumaka Island.

The ratio of marked to unmarked pups in each recapture sample was input into a modified Petersen estimate [61] as follows:

$$Pi=\left[\frac{\left(M+1)(Ci+1\right)}{Ri+1}\right]-1$$

For each replicate *i*, *M* is the number of marked fur seal pups, *Ci* represents the total number of pups in the recapture sample and *Ri* is the number of marked pups in the recapture sample. Consequently, the total estimate of live pups, *P*, is calculated from the mean of the *Q* Petersen estimators as:

$$P=\frac{\sum_{i=1}^{Q}Pi}{Q}$$

The standard error (SE) of *P* was calculated from the individual estimates [62] as:

$$SE=\sqrt{\frac{1}{Q\left(Q-1\right)}\sum_{i=1}^{Q}{\left(Pi-P\right)}^{2}}$$

Counts of dead pups observed during marking have been made since 1997. To maintain a consistent methodology throughout the study, these counts are ignored for the purposes of the mark-recapture estimates and analysed separately. 

### 2.3. Pup Condition Observations

Biometric data were collected from a subset of captured pups and comprise measurements of mass (kg) and length (cm) [32,43]. Pup condition sample sizes are provided in Table S1. Pups were placed in polypropylene or light canvas weighing bags and weighed using 20 kg Pesola hook scales (precision of ±0.1 kg). A draper’s tape measure was used to record pup length. Measurements were taken from nose-tip to tail-tip with pups restrained in an outstretched position. Dates of measurement have occasionally varied over the study period (Figure S1), although surveys have mostly been concentrated in the last week of January.

### 2.4. Body Condition Index Calculations

Two different Body Condition Indices (BCI), in addition to pup mass, were adopted to assess pup condition between 1991 and 2025.

Body Condition Index 1 (BCI1) was calculated by dividing a pup’s mass by its length [19,43,63]. Body Condition Index 2 (BCI2) was derived by dividing a pup’s observed mass (ObsM) by its expected mass (ExpM) [19,32,43]. ExpM was calculated by regressing log_e_Mass against log_e_Length, and applying the regression equation to log_e_Length, where *a* and *b* are the least-squares regression coefficients [32]:

log_e_ExpM = *a* + *b ×* Log_e_Length

BCI2 results > 1 indicate that pups are in better-than-expected condition, whereas results < 1 indicate that pups are in worse-than-expected condition [32,43].

### 2.5. Statistical Analyses

#### 2.5.1. Effect of Year on Live Pup Abundances 1991/92–2025

Generalized additive models (GAMs) were used to analyse the effect of year on mean live pup abundance estimates derived from mark-recapture across the duration of the study: 1991–2025 for Cape Foulwind and Taumaka Island, and 1992–2025 for Wekakura Point. These analyses included 2012–2014 at Cape Foulwind and Taumaka Island, when data were collected by a different researcher (H. Best, unpublished data). GAMs were selected to allow for non-linear effects of year on pup abundance [64] and were fitted using the *mgcv* package [65] in R (version 4.4.3) [66]. A Gaussian distribution with an identity link function was assumed for Cape Foulwind and Taumaka Island, whereas for Wekakura Point a gamma distribution with a log link function was assumed.

Derivatives can be used to identify important moments of change in GAMs being used to assess temporal trends [64,67]. Here, change in the sign (positive/negative) of the first order derivative (the gradient) of the smooths was used to identify when the trajectory of pup abundance changed from positive to negative, or vice versa (‘turning points’). The derivatives were calculated using the *gratia* package [68], and significance was assessed by determining whether the 95% confidence interval of the relevant derivative included zero [64,67].

To determine whether the rates of annual change in live pup abundances had varied between the period studied by Roberts and Neale (1991/2–2016) [57] and the current study (2016–2025), annual average percentage changes in pup abundance were calculated for each colony in both periods.

#### 2.5.2. Pan-Colony Pup Mass and Condition Models

Using pup condition data from all three colonies (‘pan-colony models’), linear models were fitted to pup mass and BCI1 in R to predict these measurements in response to a suite of explanatory variables: Year, Sex, Colony, Day of measurement (as well as interactions between these variables). The variables were included in a global model, and second-order Akaike’s Information Criterion (AICc) was used to select a preferred model structure, using the *MuMIn* package version 1.48.4 [69]. Model structures were not considered if they would involve large numbers of parameters, to avoid overfitting. BCI2 was used to compare pup condition from all colonies in all years [32].

#### 2.5.3. Individual Colony Pup Mass and Condition Models

To compare mass and condition trends over time for each colony, the respective preferred model structures for mass and BCI1 were retained, with the variable ‘Colony’ removed as a predictor. Due to a low spread of days at some colonies, in particular at Taumaka Island (Figure S1), the day effect was fixed to the values obtained from the pan-colony models. Inter-survey percentage change in model standardised mass and BCI1 results was used to evaluate the extent of change in pup mass and condition between surveys. Relationships between pup mass and pup abundance were assessed through correlation analysis and scatterplots.

## 3. Results

### 3.1. Live Pup Abundance Estimates

Live fur seal (*Arctocephalus forsteri*) pup abundance estimates between 2018 and 2025 are shown in Table 1.

**Figure 2:**
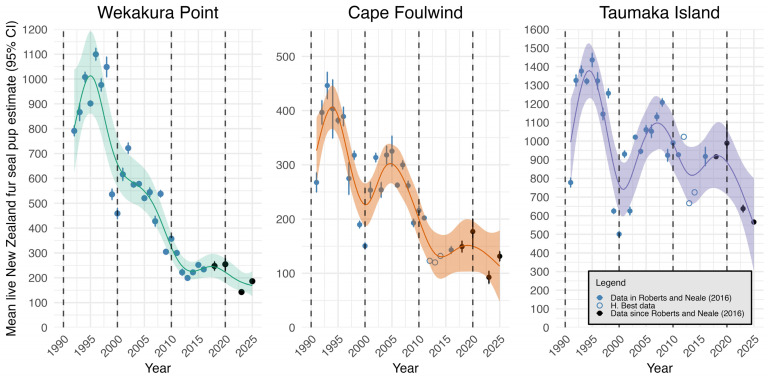
Mean live pup abundance estimates from mark-recapture at the three WCSI colonies, with smooths from the GAMs of pup estimate against year overlaid. Hollow points are used for years from Cape Foulwind and Taumaka Island when data were collected without error ranges (H. Best). The shaded areas represent the 95% confidence interval [57].

Between 2016 (the last year of Roberts and Neale [57]) and 2018, pup production was largely stable at all colonies (Figure 2). It then increased somewhat between 2018 and 2020, before dropping sharply between 2020 and 2023. Live pup abundance at Wekakura Point and Cape Foulwind increased from 2023 to 2025 but continued to fall at Taumaka Island.

### 3.2. Effects of Year on Live Pup Abundance Estimates

The results of the GAMs exploring the impact of year on live pup abundance are plotted in Figure 2 and presented in Table 2. In each case the models explained a substantial proportion of the deviance, and the smooth terms for year were highly significant.

Turning points in the trajectory of live pup abundance estimates at Wekakura Point were identified in 1995/1996 (positive to negative), 2014 (negative to positive) and 2017/2018 (positive to negative) (Figure 2). The 95% confidence intervals showed that 1995/1996 was the only turning point when the associated derivative was significantly different from zero. Indeed, since 2012, the only period where the gradient of the GAM smooth was significantly different from zero was in 2021/2022, when the gradient was significantly negative.

Turning points in the trajectory of live pup abundance at Cape Foulwind were identified in 1994/1995 (positive to negative), 1999/2000 (negative to positive), 2005/2006 (positive to negative), 2014/2015 (negative to positive) and 2018/2019 (positive to negative). First-order derivatives showed significant positive gradients between 1991 and 1993 and 2001–2003, and significant negative gradients between 1994 and 1999 and 2007–2012. Since 2012, the gradient for Cape Foulwind has been non-significant.

Turning points in the trajectory of live pup abundance at Taumaka Island were identified in 1994/1995 (positive to negative), 2000/2001 (negative to positive), 2007/2008 (positive to negative), 2014/2015 (negative to positive) and 2018 (positive to negative). First-order derivatives showed significant positive gradients between 1991 and 1994 and 2002–2005 and significant negative gradients between 1995 and 2000, 2009–2011 and 2021–2023.

Comparisons of percentage change in live pup abundance estimates from each colony’s recorded maximum to 2016 (the final year of Roberts and Neale [57]) and 2016–2025 are presented in Table 3.

### 3.3. Dead Pup Counts

**Figure 3:**
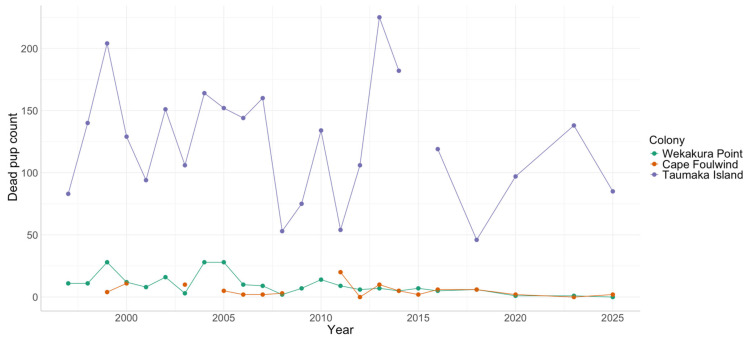
Counts of dead New Zealand fur seal pups at the Wekakura Point, Cape Foulwind and Taumaka Island colonies between 1997 and 2025.

Dead pup counts have been consistently much higher at Taumaka Island than at Wekakura Point or Cape Foulwind (Table S2) and this pattern has continued between 2016 and 2025 (Figure 3). On average, since dead pup counts started in 1997, dead pup counts as a percentage of total pups (live plus dead) has been 12.7% (range 4.2–25.2%) at Taumaka Island, compared to 2.3% (range 0–5.4%) at Wekakura Point and 3% (0–9%) at Cape Foulwind.

Sharp declines in the live pup abundance estimates in 2023 (Table 1) coincided with a high dead pup count at Taumaka Island, but not at Wekakura Point and Cape Foulwind, where dead pup counts remained low. The 2018 dead pup count at Taumaka Island was the lowest ever recorded for that colony.

### 3.4. Pup Size and Condition

Between 1991 and 2025, mean pup mass at Wekakura Point was 6.68 kg (range = 3–13 kg), and mean BCI 1 was 0.09 kg/cm (range = 0.05–0.17 kg/cm). At Cape Foulwind, mean pup mass was 6.88 kg (range = 3–11.8 kg) and mean BCI1 was 0.1 kg/cm (range = 0.04–0.17 kg/cm). At Taumaka Island, mean pup mass was 6.12 kg (range = 2.16–11.4 kg) and mean BCI1 was 0.09 kg/cm (range = 0.04–0.14 kg/cm).

**Figure 4:**
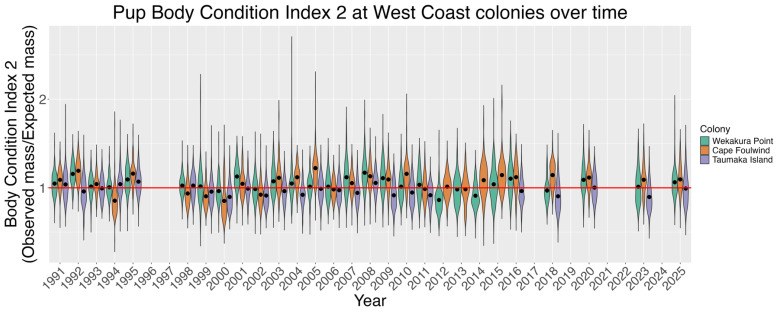
BCI2 values for fur seal pups sampled at Wekakura Point, Cape Foulwind and Taumaka Island, derived from a comparison of all pups at all colonies in all years. Violin shapes show the frequency of values, and black dots denote annual means. Values above 1 denote pups in better-than-expected condition, and values below 1 denote pups in worse-than-expected condition.

BCI2 was used to compare the pups from all colonies in all years (Figure 4). BCI2 values greater than 1 indicate pups were in better-than-expected condition, whereas values less than 1 indicate pups were in worse-than-expected-condition. On average, Wekakura Point pups were in better-than-expected condition in 22 out of 28 years, and in worse-than expected condition in 6. On average, Cape Foulwind pups were in better-than-expected condition in 20 out of 28 years, and in worse-than-expected condition in 8. On average, Taumaka Island pups were in better-than-expected condition in 6 out of 24 years, and in worse-than-expected condition in 18.

#### 3.4.1. Pan-Colony Pup Mass and Condition Model Results

Results of the AIC(c) model selections are in Tables S3 and S4.

The preferred pan-colony mass model (adj. r^2^ = 0.22, F_33,12377_ = 109.9, *p* < 0.001) included Colony, Day, Sex and Year. On average, Taumaka Island pups were 0.75 kg (95% CI = 0.69–0.81 kg, *p* < 0.001) lighter than Cape Foulwind pups and Wekakura Point pups were 0.14 kg lighter than Cape Foulwind pups (95% CI = 0.08–0.2 kg, *p* < 0.001). Pup mass increased by 0.04 kg per day (95% CI = 0.03–0.05 kg, *p* < 0.001), and male pups were 0.69 kg heavier than female pups (95% CI = 0.64–0.73 kg, *p* < 0.001). Most years were significantly different (*p* < 0.05) from the model baseline with full model results shown in Table S5.

The preferred BCI1 model (adj. r^2^ = 0.21, F_30,11,576_ = 104.4, *p* < 0.001) included Colony, Sex and Year. On average, Taumaka Island pups were 0.01 kg/cm lighter than Cape Foulwind pups (95% CI = 0.009–0.01 kg/cm, *p* < 0.001), and Wekakura Point pups were 0.002 kg/cm lighter than Cape Foulwind pups (95% CI = 0.001–0.003 kg/cm, *p* < 0.001). Male pups were 0.01 kg/cm heavier than females (95% CI = 0.006–0.007 kg/cm, *p* < 0.001). Most years were significantly different (*p* < 0.05) from the model baseline with full model results shown in Table S6.

#### 3.4.2. Individual Colony Pup Mass and Condition Model Results

**Figure 5:**
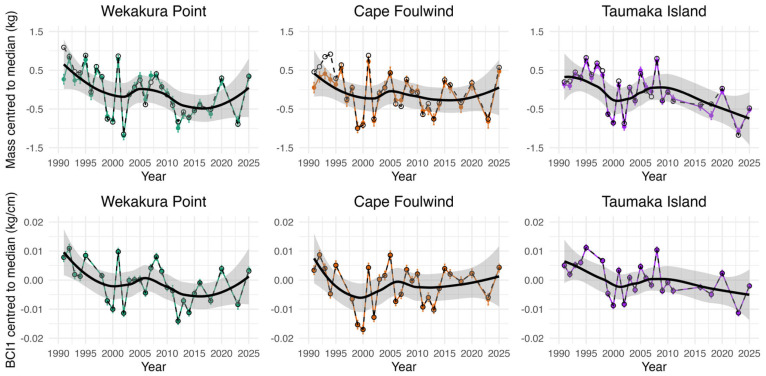
Annual average pup mass and BCI1 (dashed lines) and model standardised estimates with standard error bars (coloured lines) for the three WCSI study colonies. Both non-standardised and standardised estimates have been centred to median, and LOESS smoothers (span = 0.75) have been applied to aid trend visualisation.

The individual colony linear models showed that male pups had significantly higher mass and BCI1 than female pups (*p* < 0.001), and most years were significantly different from the baseline for each colony (*p* < 0.05). Annual average pup masses and BCI1, as well as model standardised estimates, are presented in Figure 5. Model standardised mass estimates were typically close to raw values, with deviations occurring in years when pups were weighed later in the season—for example, 1991 at Wekakura Point, 1991–1994 at Cape Foulwind and 2018 at Taumaka Island.

Using the model standardised estimates, inter-survey percentage change for mass and BCI1 was calculated to determine when the largest changes occurred (Table S7).

**Figure 6:**
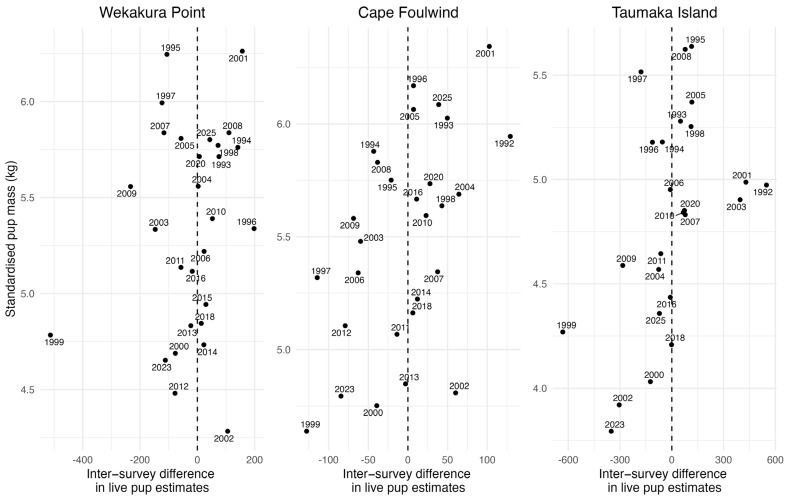
Pairwise scatterplots showing relationships between model standardised fur seal pup mass and the inter-survey difference in live pup abundance at the three WCSI study colonies.

Pearson’s product-moment correlations found a weak, non-significant correlation between standardized pup mass and the inter-survey difference in live pup abundance estimates at Wekakura Point (r = 0.19, *p* = 0.32) and moderate, significant correlations between these variables at Cape Foulwind (r = 0.51, *p* < 0.01) and Taumaka Island (r = 0.49, *p* < 0.05). These relationships are shown in Figure 6.

## 4. Discussion

Through a consistently executed long-term monitoring programme, this study demonstrates continued declines in live pup abundance at three New Zealand fur seal (*Arctocephalus forsteri*) breeding colonies on the West Coast of New Zealand’s South Island. These declines now total 83% at Wekakura Point, 71% at Cape Foulwind and 61% at Taumaka Island from each respective recorded maximum in the early/mid-1990s. Over this period, pup condition has been highly variable, with correlations between the trajectories of pup condition and pup abundance.

The sustained declines in pup abundance at the colonies monitored in this study contrast with recent findings on the East Coast of New Zealand’s South Island [59,60], and more broadly with population trends observed in New Zealand fur seal colonies elsewhere in New Zealand, where colony sizes have largely been stable or increasing [7,44,70,71]. However, in other pinniped species, there are recent records of declines among previously growing populations [11,12,13].

The longitudinal nature of this dataset enables the detection of both consistencies and nuances in the trends across the colonies. For example, at all three colonies there were troughs in pup abundance around 2000, 2010–2012 and 2023, with relative peaks in the mid-1990s, around 2005 and 2016–2020. Wekakura Point and Cape Foulwind produced their lowest ever live recorded pup estimates in 2023: 143 (95% CI = 133–152) and 93 (95% CI = 81–104), respectively. Taumaka Island’s 2025 estimate of 566 (95% CI 555–577) is the second lowest ever recorded after 500 (95% CI = 486–516) in 2000. Future data collection is required to determine whether the declines in 2023 and 2025 relative to 2020 will be followed by increases, as previously observed, or whether the populations will continue to decline towards zero.

Since 2012, there have been more gradual rates of population change at Cape Foulwind and Wekakura Point relative to Taumaka Island. Taumaka Island is also the only colony where the rate of population change has become more negative since 2016 [57] (Table 3), and where pup mortality has been consistently high relative to the other two.

That male pups were heavier and had higher BCI1 than females, and that pups were heavier and higher BCI1 when measured later in the season conforms to both expectations and past studies [43,72,73]. On average, Cape Foulwind pups were the heaviest and had the highest BCI1, followed by Wekakura Point and then Taumaka Island. From BCI2, Wekakura Point and Cape Foulwind pups were in better-than-expected condition in most years, whereas Taumaka Island pups were in worse-than-expected condition in most years (Figure 4). Pup mass and BCI1 have varied substantially at the three colonies through time, with consistencies in their temporal trends (Figure 5). There were also correlations between declines in live pup abundance estimates and decreases in pup condition, including in recent years. For example, between 2020 and 2023 average pup mass decreased by 16.4–21.8% at the three study colonies at the same time as they experienced their largest inter-assessment decreases in live pup abundance estimates since 2016. Average pup mass then increased substantially (14.9–26.9%) at all colonies from 2023 to 2025. At Wekakura Point and Cape Foulwind live pup abundance estimates also increased, but continued to decrease at Taumaka Island.

### 4.1. Potential Causes of the Colony Declines

Establishing causation for long-term population declines in pinnipeds is complex due to the variety of potential drivers [13,24,25]. WCSI live pup abundance estimates between 1991 and 2025 demonstrate both episodic drops, sometimes followed by partial recoveries (Figure 2), and long-term declines.

#### 4.1.1. Episodic Drops in Pup Abundance

Unless associated with low pup survival [74,75], episodic drops in pup numbers are typically a reflection of low pup production [76,77]. Reductions to pinniped pup production can result from direct mortality of females—for example, through bycatch in fisheries [33,78]—but also through environmentally mediated factors that impact fecundity. For example, due to the energetic costs associated with gestation and pup rearing, nutritionally stressed females may fail to implant following embryonic diapause [28] or abort in late gestation [79,80]. Changing oceanographic conditions, such as increases to sea surface temperature (SST), can engender changes to pinniped prey location and reliability, forcing lactating females to expend varying levels of energy when foraging [18,81,82], which may then impact fecundity [80,83]. Such a relationship may explain why sharp drops in pup production at all three colonies between 1998 and 2000 and, to a lesser extent, around 2010, aligned with strong, warm La Niña events [84], as warmer waters have been associated with poorer foraging conditions in several pinnipeds [18,82]. Roberts et al. (2022) [84] found that WCSI fur seal productivity was reduced in years with elevated SST, when the surface chlorophyll-a (chl-a) concentration was low, the latter driven by shallowing of the mixed layer depth. Intense warm extremes in the New Zealand region have occurred between November–March in 2017/2018, 2018/2019, 2021/2022 and 2022/2023, with the highest marine heatwave anomalies occurring off the WCSI [85]. During this period (2016–2023), the overall trend at the WCSI colonies has been towards lower pup abundance. Given the findings of Roberts et al. (2022) [84], and correlations between high WCSI SST and low fur seal productivity on the WCSI between 2018 and 2023, it seems likely that changing marine environmental conditions are at least partly contributing to the observed declines in WCSI pup abundance at these colonies.

Disease can also impact fecundity [20,48], as well as survival [16,86,87] in pinnipeds. Recently, a divergent strain of canine distemper virus (CDV) was detected in Kaikōura’s New Zealand fur seals through consistent colony monitoring [88]. This pathogen was associated with an Unusual Mortality Event and substantially reduced pup production at the region’s largest colony as well as high observed rates of starvation and nutritional stress (Hall et al. *in prep*). CDV has subsequently been detected at Mātakitaki-a-Kupe/Cape Palliser, near Wellington, the largest recorded fur seal colony on New Zealand’s North Island (Taylor et al. *in prep*), again in association with elevated observed mortality. Only a limited number of necropsies have been performed at the WCSI colonies over the study period, and future monitoring should incorporate disease screening, so that the potential contribution of CDV or other pathogens to the recorded declines can be determined.

#### 4.1.2. Long-Term Declines in Breeding Colony Sizes

In addition to the abrupt drops in pup abundance there have also been protracted declines, which are more likely to reflect reduced survival across multiple female age classes [19,89,90], or emigration (see below), than reduced fecundity or pup survival. Pinniped survival can be impacted by numerous non-mutually exclusive factors including disease [16,88,91], legal or illegal anthropogenic killings [92,93,94], bycatch mortality [22,95,96,97], entanglement [23,98], interactions with human infrastructure such as roads and oil extraction and transportation activities [60,99,100], injuries from aggressive in-colony con-specific encounters [101,102], predation [12,103,104] and starvation as a result of changes to prey availability [105].

On the WCSI, predation can be discounted as a substantial cause of pup mortality during the surveying window, as New Zealand fur seals lack terrestrial predators, and pups at this stage are on land or in shallow pools around the colony. Not enough is known about predation of adult fur seals on the WCSI, so this cannot be discounted. The long-term nature of the pup abundance declines means that fatal in-colony encounters between fur seals is also an unsatisfactory explanation, as the frequency of such encounters should reduce as colony density decreases [106]. On the East Coast of the South Island, fur seal colony accessibility has been associated with illegal killings [94] and roadkill mortality [99]. By contrast, the three WCSI study colonies are much harder to access, meaning terrestrial human activities are less likely to impact fur seal survival. At sea, however, New Zealand fur seals suffer high rates of incidental capture and mortality [97,107,108]. Between 2002/03 and 2022/23, 2044 fur seals were reported captured in trawl fisheries in New Zealand waters, and a further 517 in surface longline fisheries [109], with the WCSI known as a hotspot for fur seal bycatch [34]. Thus, WCSI fur seal survival is impacted directly by bycatch in trawl, set and surface longline fisheries. Due to reduced fur seal monitoring frequency at the WCSI colonies since 2016, some precision has been lost when assessing potential relationships between fur seal bycatch and population trends, particularly given the substantial assessment-to-assessment variability in live pup abundance estimates. Preferably, monitoring should return to an annual basis.

As well as potentially impacting WCSI fur seal fecundity, nutritional stress may also impact local fur seal survival. There are links between condition and both pre- and post-weaning survival in pinniped pups [29,56], which then impact future population sizes in a lagged effect due to lower recruitment [19,98]. As pup condition can be used as a proxy for female foraging success [18,19], low pup condition likely indicates concomitant nutritional stress in females that could reduce survival in this cohort as well [105]. Adult females are particularly important to pinniped population trajectories [110,111,112], and their status as central-place foragers [113,114], means features of the local marine environment can drive inter-colony differences in condition [17,115].

The combination of considerably higher pup mortality and significantly lower condition (mass and BCI1) at Taumaka Island relative to the other colonies may indicate that Taumaka Island declines are being influenced primarily by poor pup survival. Contrastingly, lower pup mortality at Wekakura Point and Cape Foulwind could indicate that their declines are being influenced more by lower pup production (either through declining female survival or low breeding rates) than poor pup survival, as has been suggested for California sea lions (*Zalophus californianus*) at colonies in the Gulf of California [18]. Importantly, both poor pup survival and low pup production could relate to prey availability, as the timing of prey scarcity can influence both otariid pup production and pup survival rates [18,105]. Comparative analysis of foraging habits [116,117,118] between females from the WCSI colonies, combined with scat sampling [119,120] and isotopic analysis of vibrissae and/or pup fur [18,121] would benefit understandings of the relative ease with which females from the three colonies can find prey, as well as elucidating the prey items targeted and the trophic level(s) at which they are foraging.

Despite the nuances, both poor survival and occasional abrupt pup production decreases have likely impacted all three colonies, as all have experienced both long-term declines and episodic drops, and similarities in the trends at the three colonies are consistent with shared drivers [84] (Figure 2). If, as seems likely, reduced female foraging efficiency (linked to prey availability) is among these drivers [51,84], changes to colony densities through time might explain some of the variation in their rates of decline. Effects of reduced foraging efficiency should be at least somewhat density dependent, with fewer resources per individual at larger colonies [18,32]. Fur seal abundance at Taumaka Island initially decreased more slowly than at the northern colonies, and this colony remains substantially larger in terms of pup production. Thus, a bottom-up limitation from reduced female foraging efficiency may now act more strongly on the Taumaka Island colony than either of the others [18], perhaps partially explaining why the former is now declining more rapidly.

Migration out of the study area could also engender the observed reductions in WCSI pup abundance, as has been observed in Cape fur seals (*Arctocephalus pusillus*) in Southern Africa [3]. As this research focuses only on the three study colonies, this cannot be quantitatively assessed. While there have been suggestions that some Wekakura Point fur seals might have relocated to other colonies in the surrounding area (L. Angus, pers. comm.), no data have been collected to support this, and there is no suggestion of a similar phenomenon at either Cape Foulwind or Taumaka Island. Limited fur seal monitoring in New Zealand makes it hard to assess such claims [41], and large-scale surveying would be required to detect such movement [3,30]. The last time that the entire WCSI region was surveyed was by helicopter in January 2009 [122].

### 4.2. Recommendations for Future Monitoring and Research

#### 4.2.1. Abundance and Health Monitoring

Ideally, WCSI fur seal monitoring should revert to an annual basis to avoid masking interannual variability. As the WCSI monitoring programme represents one of two longitudinal fur seal population studies in New Zealand, and the only one to include condition monitoring, this work is important for broader understandings of the species in New Zealand. This is particularly important in the context of threats such as Highly Pathogenic Avian Influenza (HPAI)—which has been associated with catastrophic mortality in pinniped species in the Southern Hemisphere [14,16], and is getting closer to New Zealand—and the effects of climate change on marine ecosystems [23,123]. Pinnipeds can be effective sentinels for threats such as new diseases [88,124] and environmental change [82,125], and continued monitoring would enable the use of fur seal abundance and body condition measurements to detect changes, such as reduced food availability (pup abundance and body condition) or novel pathogens (pup abundance and survival). The results of future monitoring of pup condition and abundance should also be assessed in context with rates of fisheries bycatch and mortality through time, as another known source of mortality to fur seals in the region.

#### 4.2.2. Diet and Foraging Studies

Updated research on WSCI fur seal diet and foraging could elucidate how these parameters may be contributing to the trajectories outlined in this study. Routine scat and regurgitate sampling can be effective for understanding the biomass contributions of different prey species to fur seal diet [120]. This, combined with satellite telemetry studies of foraging behaviour and the location of important foraging grounds for lactating female fur seals would provide much needed spatial and temporal data to inform models seeking to understand and mitigate risks to the species [107].

### 4.3. Conclusions

The data presented here demonstrate continued declines at three New Zealand fur seal colonies on the West Coast of New Zealand’s South Island, as well as highly variable pup condition. This contrasts with most recent population studies of the species in New Zealand, where other monitored colonies appear to be growing or stable.

Given the temporal consistencies between pup production and pup body condition trends at the WCSI colonies, and previously recorded links between fur seal pup productivity and climate drivers [84], it seems likely that marine environmental change is playing a role in the trends observed here. However, in the context of the recent discovery of CDV in New Zealand fur seals [88] and the particularly high number of direct mortalities in fisheries [34,108], it is important that a range of potential drivers continue to be assessed, and mitigation options implemented where possible.

## Figures and Tables

**Table 1:** Summary of the methods and results from New Zealand fur seal pup mark-recapture (2018–2025) at the three study colonies on the West Coast of the South Island of New Zealand.

Colony	Year	Dates of Marking	No. Pups Marked	Dates of Recapture	Mean Mark-Recapture Estimate (95% Confidence Interval)
Wekakura Point	2018	30–31 January	150	31 January–2 February, 27 February	248 (230–265)
	2020	27–28 January	106	29–30 January	254 (231–278)
	2023	24–26 January	93	25–27 January	143 (133–152)
	2025	28–29 January	135	29–31 January	186 (178–194)
Cape Foulwind	2018	30–31 January	118	7–9 February	149 (139–160)
	2020	28–29 January	129	30 January–31 January, 11 February	177 (154–200)
	2023	26–27 January	60	1–3 February	93 (81–104)
	2025	28–30 January	89	31 January–2 February	131 (122–140)
Taumaka Island	2018	4–5 February	350	6–7 February	916 (910–922)
	2020	27–30 January	384	31 January–1 February	989 (888–1089)
	2023	22–25 January	368	26–27 January	638 (619–657)
	2025	27–29 January	357	30–31 January	566 (555–577)

**Table 2:** GAM results showing relationships between Year and Live Pup Abundance at each colony.

Colony	Adjusted R^2^	Deviance Explained	Smooth Year Term Summary
Wekakura Point	0.883	93.8%	edf = 7.21, F = 41.59, *p* < 0.001
Cape Foulwind	0.853	89.6%	edf = 8.18, F = 18.68, *p* < 0.001
Taumaka Island	0.669	75.9%	edf = 7.63, F = 6.89, *p* < 0.001

**Table 3:** Percentage change in live New Zealand fur seal pup abundances at the WCSI colonies comparing the declines between the maximum recorded at each colony to 2016 (the end of the period studied by Roberts and Neale) with the data collected between 2016 and 2025.

Colony	Period	Percentage Change (%)
Wekakura Point	1996–2016	−78.7
	2016–2025	−20.5
Cape Foulwind	1993–2016	−67.9
	2016–2025	−8.4
Taumaka Island	1995–2016	−36
	2016–2025	−38.3

## Data Availability

The datasets analysed during the current study are available from the corresponding author on reasonable request. Data collected during surveys of New Zealand fur seal at the study colonies can be found at https://furseals.dragonfly.co.nz/ (URL accessed on 3 March 2025).

## Supplementary Materials

The following supporting information can be downloaded at: https://www.mdpi.com/article/10.3390/ani16010121/s1, Figure S1: Mean date (Julian date) of pup measurements used in the analyses of the WCSI study colonies through time; Table S1: Sample sizes used for analyses of pup mass. BCI1 and BCI2 sample sizes are equivalent unless stated otherwise in brackets, except for 1996 and 1997 (*), when no length data were collected meaning BCI1 and BCI2 could not be calculated; Table S2: Counts of dead pups by colony from the three WCSI study colonies, with live numbers calculated from mark-recapture, and dead pups as a percentage of the total (live + dead); Table S3: Comparison of models considered for predicting mass of pups born at WCSI fur seal colonies. Models are displayed in ascending order of AICc. Only the first 10 models are provided; Table S4: Comparison of models considered for predicting pup BCI1 born at WCSI fur seal colonies. Models are displayed in ascending order of AICc. Only the first 10 models are provided; Table S5: Results of the preferred pan-colony pup mass model; Table S6: Results of the preferred pan-colony pup BCI1 model; Table S7: Inter-survey changes in model standardised pup mass and pup BCI1 at the WCSI colonies For concision, only changes >10% are shown.
