# Supplementary material for: Declining Abundance and Variable Condition of Fur Seal (Arctocephalus forsteri) Pups on the West Coast of New Zealand’s South Island
Source: Animals (Basel). 2025 Dec 31;16(1):121. doi: 10.3390/ani16010121 (PMC12785031; doi:10.3390/ani16010121)
Supplement: Supplementary file 1 [file animals-16-00121-s001.zip › animals-4022101-supplementary.pdf]

Supplementary Materials

Table S1 Sample sizes used for analyses of pup mass. BCI1 and BCI2 sample sizes are equivalent unless stated otherwise in brackets, except for 1996 and 1997 (*), when no length data were collected meaning BCI1 and BCI2 could not be calculated.

Year	Wekakura Point	Cape Foulwind	Taumaka Island
1991	100	100	202
1992	100	100	220 (219)
1993	100	100	208
1994	100	100	211
1995	100	100	200
1996*	100	100	198
1997*	100	100	200
1998	299	150	383
1999	100	100	204
2000	150 (149)	101	202
2001	150	100	202
2002	150	100	200
2003	150	100	199
2004	150	100	200
2005	150	100	200
2006	150	100	200
2007	150	101	201
2008	150	99 (97)	200
2009	150	102	202
2010	150	100	204
2011	58	100	200
2012	150	98	-
2013	148	90	-
2014	150	99	-
2015	150	100	-
2016	150	100	200 (199)
2018	150	99	149 (148)
2020	106	101	200
2023	92	60	200
2025	134	89	200

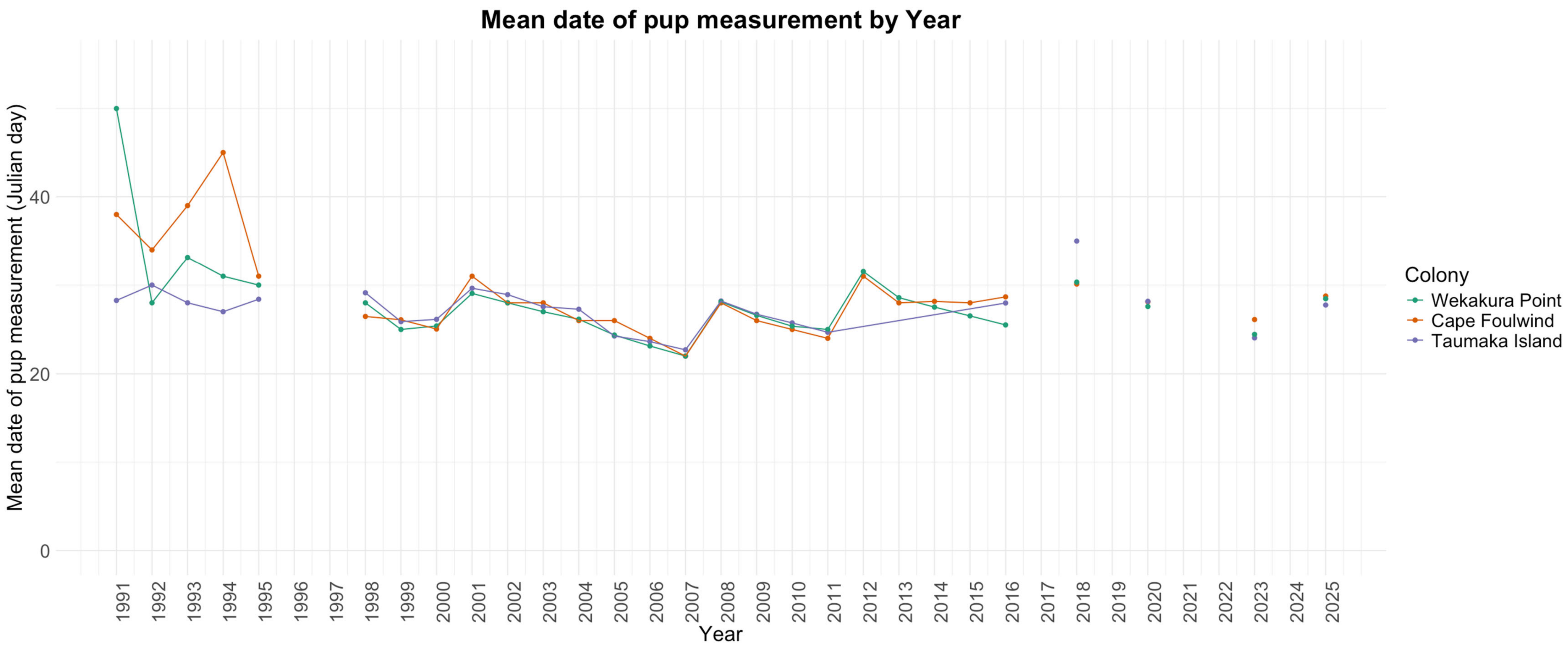

Figure S1 Mean date (Julian date) of pup measurements used in the analyses of the WCSI study colonies through time.

Table S2 Counts of dead pups by colony from the three WCSI study colonies, with live numbers calculated from mark-recapture, and dead pups as a percentage of the total (live + dead).

Year	Wekakura Point			Cape Foulwind			Taumaka Island		
	Dead	Alive	Dead as % of total	Dead	Alive	Dead as % of total	Dead	Alive	Dead as % of total
1997	11	976	1.1	NA	275	NA	83	1144	6.7
1998	11	1048	1.1	NA	318	NA	140	1257	10
1999	28	536	5.2	4	190	2.1	204	625	24.6
2000	12	459	2.6	11	151	6.8	129	501	20.5
2001	8	616	1.3	NA	253	NA	94	930	9.2
2002	16	722	2.2	NA	313	NA	151	625	19.5
2003	3	575	0.5	10	254	3.8	106	1021	9.4
2004	28	578	4.8	NA	318	NA	164	945	14.9
2005	28	521	5.4	5	325	1.5	152	1061	12.5
2006	10	544	1.9	2	262	0.8	144	1053	12
2007	9	428	2.1	2	300	0.7	160	1130	12.4
2008	2	538	0.4	3	262	1.1	53	1208	4.2
2009	7	305	2.3	NA	193	NA	75	924	7.5
2010	14	357	3.9	NA	216	NA	134	990	11.9
2011	9	300	3	20	202	9	54	926	5.5
2012	6	222	2.7	0	123	0	106	1023	9.4
2013	7	199	3.5	10	120	7.8	225	667	25.2
2014	5	222	2.2	5	132	3.7	182	725	20
2015	7	252	2.8	3	NA	NA	NA	NA	NA
2016	5	234	2.1	6	143	4	119	918	11.5
2018	6	248	2.4	6	149	3.9	46	916	4.8
2020	1	254	0.4	2	177	1.1	97	999	8.9
2023	1	143	0.7	0	93	0	138	638	17.8
2025	0	186	0	2	131	1.5	85	566	13.0
Avg (%)			2.3			3			12.7
Range (%)			0 – 5.4			0 – 9			4.2 – 25.2

Table S3 Comparison of models considered for predicting mass of pups born at WCSI fur seal colonies. Models are displayed in ascending order of AICc. Only the first 10 models are provided.

(Intercept)	colony	julian_ day	sex	Year	colony: sex	julian_ day: sex	df	logLi	AICc	delta	weight
5.77	+	0.04	+	+	NA	NA	35	-20526.13	41122.45	0.0000	0.55
5.83	+	0.03	+	+	NA	+	36	-20525.91	41124.03	1.58	0.25
5.77	+	0.04	+	+	+	NA	37	-20525.55	41125.32	2.86	0.13
5.83	+	0.03	+	+	+	+	38	-20525.30	41126.84	4.39	0.06
7.09	+	NA	+	+	NA	NA	34	-20553.25	41174.68	52.23	2.52e-12
7.09	+	NA	+	+	+	NA	36	-20552.68	41177.57	55.11	6e-13
4.38	NA	0.06	+	+	NA	NA	33	-20908.15	41882.48	760.02	5.08e-166
4.43	NA	0.06	+	+	NA	+	34	-20908	41884.19	761.74	2.16e-166
6.13	+	0.04	NA	+	NA	NA	34	-20969.69	42007.57	885.12	3.499e-193
6.64	NA	NA	+	+	NA	NA	32	-20991.27	42046.70	924.25	1.11e-201

Table S4 Comparison of models considered for predicting pup BCI1 born at WCSI fur seal colonies. Models are displayed in ascending order of AICc. Only the first 10 models are provided.

(Intercept)	colony	julian_ day	sex	Year	colony: sex	julian_ day: sex	df	logLik	AICc	delta	weight
0.1	+	NA	+	+	NA	NA	32	32636.16	-65208.13	0.00	0.49
0.1	+	-4.58E-05	+	+	NA	NA	33	32636.50	-65206.80	1.33	0.25
0.1	+	-2.97E-05	+	+	NA	+	34	32636.60	-65205.00	3.14	0.1
0.1	+	NA	+	+	+	NA	34	32636.43	-65204.65	3.48	0.09
0.1	+	-4.59E-05	+	+	+	NA	35	32636.77	-65203.32	4.82	0.044
0.1	+	-3.26E-05	+	+	+	+	36	32636.84	-65201.45	6.69	0.017
0.1	+	NA	NA	+	NA	NA	31	32347.30	-64632.43	575.70	4.80e-126
0.1	+	-4.60E-05	NA	+	NA	NA	32	32347.63	-64631.07	577.07	2.43e-126
0.08	NA	0	+	+	NA	NA	31	32177.03	-64291.88	916.25	5.39e-200
0.08	NA	0	+	+	NA	+	32	32177.18	-64290.18	917.95	2.31e-200

Table S5 Results of the preferred pan-colony pup mass model.

	Estimate	Std. Error	t value	Pr(>|t|)
(Intercept)	5.7744632	0.1910627	30.2228701	1.16753620442438e-193
colonyTaumaka Island	-0.7528959	0.03023478	-24.901651	1.34509605587988e-133
colonyWekakura Point	-0.1420106	0.03094473	-4.5891688	4.49390011195075e-06
julian_day	0.03557124	0.00483115	7.36289352	1.91354131542791e-13
sexM	0.68867916	0.02274115	30.2833954	2.11832248970039e-194
Year1992	0.17364088	0.09241962	1.87883133	0.06029101
Year1993	0.21524149	0.09121481	2.35972073	0.01830413
Year1994	0.14011622	0.0906558	1.54558467	0.12223047
Year1995	0.45748599	0.09503299	4.81397055	1.49710098793152e-06
Year1996	0.10559733	0.09590295	1.10108524	0.27088097
Year1997	0.22545004	0.0972391	2.31851225	0.02043771
Year1998	0.13236312	0.08574522	1.54367922	0.12269163
Year1999	-0.8689457	0.10234409	-8.4904336	2.29218947449773e-17
Year2000	-0.9810196	0.10042958	-9.7682336	1.85765933880279e-22
Year2001	0.31985092	0.09211686	3.47222985	0.00051792
Year2002	-1.1534639	0.09455741	-12.198556	4.96025314115361e-34
Year2003	-0.2179096	0.09647466	-2.2587241	0.02391788
Year2004	-0.2446407	0.09828131	-2.4891884	0.01281654
Year2005	0.27815551	0.10296799	2.70137841	0.00691468
Year2006	-0.2653975	0.10601436	-2.5034108	0.01231304
Year2007	-0.1125242	0.10946017	-1.0279919	0.30397371
Year2008	0.34891947	0.09514352	3.66729635	0.00024616
Year2009	-0.2607606	0.09837102	-2.6507863	0.00804068
Year2010	-0.200232	0.10086324	-1.9851828	0.04714617
Year2011	-0.4842025	0.10764769	-4.4980293	6.92105722462901e-06
Year2012	-0.968912	0.10598198	-9.1422334	7.06378247635206e-20
Year2013	-0.8563387	0.11145143	-7.6835154	1.66426018491953e-14
Year2014	-0.768862	0.11116463	-6.9164261	4.85961959279433e-12
Year2015	-0.4049461	0.11229862	-3.6059754	0.00031221
Year2016	-0.4565577	0.09676975	-4.7179795	2.40804111974256e-06
Year2018	-0.7549063	0.09183365	-8.2203676	2.23010869848769e-16
Year2020	-0.0706366	0.09727055	-0.7261873	0.4677377
Year2023	-1.1179262	0.10785143	-10.365428	4.51022808104731e-25
Year2025	-0.193222	0.09611321	-2.0103584	0.04441492

Table S6 Results of the preferred pan-colony pup BCI1 model.

	Estimate	Std. Error	t value	Pr(>|t|)
(Intercept)	0.09865357	0.00077751	126.884133	0
colonyTaumaka Island	-0.0099614	0.00035183	-28.313003	1.43088101406614e-170
colonyWekakura Point	-0.0020902	0.00036368	-5.7472903	9.29737668091636e-09
sexM	0.00657127	0.00027036	24.3054004	2.58225134240456e-127
Year1992	0.00039361	0.00101664	0.38717093	0.69863679
Year1993	-0.0012048	0.00102332	-1.1773359	0.23908568
Year1994	-0.0030304	0.00102146	-2.9667596	0.00301571
Year1995	0.00366659	0.00102838	3.56540053	0.00036477
Year1998	-0.0030155	0.00088515	-3.4068287	0.00065946
Year1999	-0.0131967	0.00102583	-12.864457	1.29084684746679e-37
Year2000	-0.0163469	0.00099857	-16.370264	1.453032071337e-59
Year2001	0.00036215	0.00099859	0.36266055	0.71686509
Year2002	-0.0156217	0.00099964	-15.62723	1.70940992573412e-54
Year2003	-0.0048829	0.00100017	-4.8820641	1.0638286208759e-06
Year2004	-0.0064247	0.00099964	-6.4270384	1.35224310911006e-10
Year2005	-0.0012746	0.00099964	-1.275063	0.20231252
Year2006	-0.0080875	0.00099964	-8.0903499	6.5421072868836e-16
Year2007	-0.0057779	0.00099859	-5.7860413	7.39320922307819e-09
Year2008	0.002789	0.00100124	2.78555562	0.0053523
Year2009	-0.0060052	0.00099755	-6.0200229	1.7967659362297e-09
Year2010	-0.0059989	0.00099754	-6.013726	1.86777850288215e-09
Year2011	-0.010559	0.00105849	-9.9755371	2.42286930539755e-23
Year2012	-0.0158002	0.00118468	-13.337083	2.79166301225276e-40
Year2013	-0.0131452	0.00119991	-10.955227	8.5865212165931e-28
Year2014	-0.0128155	0.00118321	-10.831092	3.3108570144465e-27
Year2015	-0.0061381	0.00118176	-5.1940201	2.09296125815172e-07
Year2016	-0.006245	0.00100017	-6.2438921	4.41758877163184e-10
Year2018	-0.0098812	0.00103118	-9.5824223	1.14066356565361e-21
Year2020	-0.0026246	0.00102395	-2.5631904	0.01038404
Year2023	-0.0151787	0.00106328	-14.275279	7.63780780390947e-46
Year2025	-0.0042933	0.00101448	-4.2320195	2.3338749521697e-05

Table S7 Inter-survey changes in model standardised pup mass and pup BCI1 at the WCSI colonies For concision, only changes >10% are shown.

Colony	Positive percentage change (>10%) in mass	Negative percentage change (>10%) in mass
Wekakura Point	2000 – 2001 (33.5%)	2001 – 2002 (-31.6%)
	2023 – 2025 (24.7%)	2020 – 2023 (-18.6%)
	2002 – 2003 (24.5%)	1998 – 1999 (-17.1%)
	2018 – 2020 (17.9%)	1995 – 1996 (-14.5%)
	1996 – 1997 (12.3%)	2011 – 2012 (-12.8%)
	2006 – 2007 (11.8%)	2005 – 2006 (-10.1%)
Cape Foulwind	2000 – 2001 (33.5%)	2001 – 2002 (-24.2%)
	2023 – 2025 (26.9%)	1998 – 1999 (-17.7%)
	2002 – 2003 (14%)	2020 – 2023 (-16.4%)
	2018 – 2020 (11.1%)	1996 – 1997 (-13.8%)
		2005 – 2006 (-12%)
Taumaka Island	2002 – 2003 (25%)	2020 – 2023 (-21.8%)
	2000 – 2001 (23.7%)	2001 – 2002 (-21.4%)
	2004 – 2005 (17.5%)	1998 – 1999 (-18.7%)
	2007 – 2008 (16.4%)	2008 – 2009 (-18.4%)
	2018 – 2020 (15.3%)	
	2023 – 2025 (14.9%)	

Colony	Positive percentage change (>10%) in BCI1	Negative percentage change (>10%) in BCI1
Wekakura Point	2000 – 2001 (24.3%)	2001 – 2002 (-21%)
	2023 – 2025 (14.5%)	2020 – 2023 (-12.8%)
	2002 – 2003 (14.1%)	2011 – 2012 (-11.9%)
	2018 – 2020 (12.3%)	
Cape Foulwind	2000 – 2001 (27.6%)	2000 – 2001 (-17.3%)
	2002 – 2003 (16%)	2004 – 2005 (-15.4%)
	2023 – 2025 (12.3%)	1995 – 1998 (-12%)
	1994 – 1995 (11%)	2011 – 2012 (-11.8%)
Taumaka Island	2000 – 2001 (16.3%)	2020 – 2023 (-16%)
	2007 – 2008 (14.9%)	2008 – 2009 (-15%)
	2023 – 2025 (12.9%)	2001 – 2002 (-13.5%)
	2002 – 2003 (12.3%)	1998 – 1999 (-12.4%)
	2004 – 2005 (10.1%)	
